# Supplementary material for: ProteinShader: illustrative rendering of macromolecules
Source: BMC Struct Biol. 2009 Mar 30;9:19. doi: 10.1186/1472-6807-9-19 (PMC2672931; doi:10.1186/1472-6807-9-19)
Supplement: Additional file 1 — ProteinShader program without source code. This compressed file contains the complete ProteinShader program including associated libraries, but no source code. A README.txt file gives an overview of the ProteinShader distribution, and the index.html file in the help subdirectory has directions on getting started with the program as well as a set of tutorials. [file 1472-6807-9-19-S1.zip › ProteinShader-beta-0_9_4-binary/help/api/org/proteinshader/structure/Loop.html]

Loop (ProteinShader API)


|  |  |  |
| --- | --- | --- |
| **Overview** \| **Package** \| **Class** \| **Use** \| **Tree** \| **Deprecated** \| **Index** \| **Help** | |  |
| **PREV CLASS**   **NEXT CLASS** | **FRAMES**    **NO FRAMES**     **All Classes** |  |
| SUMMARY: NESTED \| FIELD \| CONSTR \| METHOD | DETAIL: FIELD \| CONSTR \| METHOD |  |


---


## org.proteinshader.structure Class Loop

```
java.lang.Object
  org.proteinshader.structure.Region
      org.proteinshader.structure.Loop
```

**All Implemented Interfaces:**: IDTest, Visitable

---

``` public class Loop extends Region ```

This concrete subclass of Region holds information on
the general "loop" regions found between well-defined
regions of secondary structure (helices and beta-strands).

---

**Constructor Summary**

`Loop(String loopID,
String startResidueID,
String endResidueID,
Chain chain)`
  
          Constructs a Loop.


| **Method Summary** | |
| --- | --- |
| `void` | `accept(Visitor visitor)`             Accepts a Visitor and does a callback. |
| `String` | `getLoopID()`             Returns the Loop ID. |
| `String` | `toString()`             Returns the Loop ID. |

**Methods inherited from class org.proteinshader.structure.Region**

`createSegments, getAminoAcidAfterRegion, getAminoAcidBeforeRegion, getChainID, getEndResidueID, getModelID, getSegment, getStartResidueID, getStructureID, iteratorAminoAcids, iteratorSegments, markAminoAcids, numberOfAminoAcids, numberOfSegments, processID, setSegmentAlpha, setSegmentAlphaToDefault, setSegmentRGB, setSegmentRGBToDefault, setSegmentsToAminoAcidColors, setSegmentsToRegionColor`

**Methods inherited from class java.lang.Object**

`clone, equals, finalize, getClass, hashCode, notify, notifyAll, wait, wait, wait`

**Constructor Detail**

### Loop

```
public Loop(String loopID,
            String startResidueID,
            String endResidueID,
            Chain chain)
     throws InvalidRegionException,
            InvalidIDException
```

:   Constructs a Loop.


**Method Detail**

### accept

```
public void accept(Visitor visitor)
            throws VisitorException
```

:   Accepts a Visitor and does a callback.

    :   **Specified by:**: `accept` in interface `Visitable` **Specified by:**: `accept` in class `Region`
    :   **Parameters:**: `visitor` - the Visitor to do a callback with. **Throws:**: `VisitorException` - if an error occurs while an object is being visited.

---


### getLoopID

```
public String getLoopID()
```

:   Returns the Loop ID.
    The String returned cannot be null or empty, because the
    constructor checks that this read-only attribute has at least
    one character.

    :   **Returns:**: The Loop ID as a String.

---


### toString

```
public String toString()
```

:   Returns the Loop ID.
    The String returned cannot be null or empty, because the
    constructor checks that this read-only attribute has at least
    one character.

    :   **Overrides:**: `toString` in class `Object`
    :   **Returns:**: The Loop ID as a String.


---


|  |  |  |
| --- | --- | --- |
| **Overview** \| **Package** \| **Class** \| **Use** \| **Tree** \| **Deprecated** \| **Index** \| **Help** | |  |
| **PREV CLASS**   **NEXT CLASS** | **FRAMES**    **NO FRAMES**     **All Classes** |  |
| SUMMARY: NESTED \| FIELD \| CONSTR \| METHOD | DETAIL: FIELD \| CONSTR \| METHOD |  |


---

# *Copyright © 2007-2008*
